# Anomalous plasmons in a two-dimensional Dirac nodal-line Lieb lattice†

**DOI:** 10.1039/d0na00759e

**Published:** 2020-12-26

**Authors:** Chao Ding, Han Gao, Wenhui Geng, Mingwen Zhao

**Affiliations:** School of Physics, State Key Laboratory of Crystal Materials, Shandong University Jinan 250100 Shandong China zmw@sdu.edu.cn

## Abstract

Plasmons in two-dimensional (2D) Dirac materials feature an interesting regime with a tunable frequency, and long propagating length and lifetime, but are rarely achieved in the visible light regime. Using a tight-binding (TB) model in combination with first-principles calculations, we investigated plasmon modes in a 2D Lieb lattice with a Dirac nodal-line electronic structure. In contrast to conventional 2D plasmons, anomalous plasmons in the Lieb lattice exhibit the unique features of a carrier-density-independent frequency, being Landau-damping free in a wide-range of wave vectors, a high frequency, and high subwavelength confinement. Remarkably, by using first-principles calculations, we proposed a candidate material, 2D Be_2_C monolayer, to achieve these interesting plasmon properties. The plasmons in the Be_2_C monolayer can survive up to the visible frequency region and propagate to large momentum transfer that has rarely been reported. The anomalous plasmons revealed in the Lieb lattice offer a promising platform for the study of 2D plasmons as well as the design of 2D plasmonic materials.

## Abstract 2

Plasmons in two-dimensional (2D) Dirac materials feature an interesting regime with a tunable frequency, and long propagating length and lifetime, but are rarely achieved in the visible light regime.

## Graphical abstract

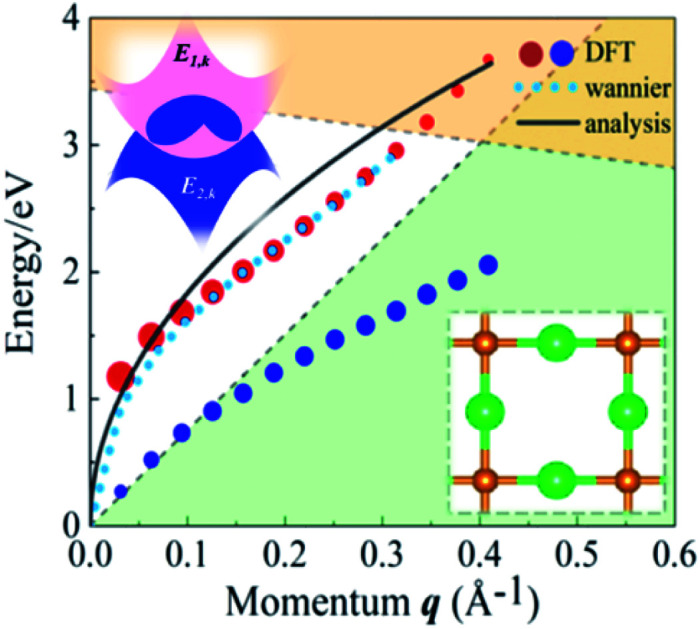

## Introduction

Plasmons, as collective excitations of electrons, enable coupling between electromagnetic radiation and electrons in materials at subwavelength scales, and a wide-range of applications, such as photodetection, biosensing and nanophotonics.^1–7^ The dimensionality of materials offers additional freedom to regulate plasmonic properties,^8–12^ among which two-dimensional (2D) materials are of particular interest, due to their unique electronic structures and quantum-confinement effects.^13–22^ For example, plasmons in graphene have been demonstrated to possess high tunability, large subwavelength confinement and a longer propagating length and lifetime both theoretically and experimentally.^2,18,19^ These plasmonic properties are closely related to the unique linear energy–momentum dispersion relation in graphene, namely Dirac cones. Additionally, the exotic electronic structures of topological materials, such as topological insulators (TI)^23^ and topological semimetal (TSM) states,^24^ offer a new platform for creating plasmons, revealing a series of interesting scenarios. Recently, a novel undamped gapless plasmon mode was predicted in a type-II Dirac semimetal, benefitting from the presence of both electron and hole pockets at the Fermi surface due to the titled Dirac cone.^25^ The plasmon properties of three-dimensional (3D) Dirac nodal line (DNL) semimetals with a nodal line Fermi ring formed by band touching points have also been investigated. In the long-wavelength limit, the plasmon frequency (*ω*_p_) shows a special dependence on the carrier density (*n*), *ω*_p_ ∝ *n*^1/4^,^26,27^ which differs significantly from the *ω*_p_ ∝ *n*^1/2^ law in 3D metals, 2D electron gas^28^ and bilayer graphene,^29^ but similar to that of graphene.^18^ The carrier-density-dependent plasmon frequency offers a simple strategy to regulate the plasmon properties, *e.g.* by doping or the gate voltage. However, it is unsuitable for applications where stable plasmons against environment perturbation are required. It is also disadvantageous for improving the plasmon density at a low frequency and the lifetime at high carrier density.^30^ Very recently, anomalous plasmons with a density-independent frequency, intensity and damping were predicted in one-dimensional topological electrides with Dirac nodal-surface states,^30^ paving a way for the design of plasmonic materials.

Another key parameter of plasmons is the maximal frequency of undamped plasmon modes, which is determined by the edges of single-particle excitation (SPE) continuum. As the collective mode enters the SPE continuum, it will be damped and decay into electron–hole pairs. Typically, plasmons in gate-doped graphene emerge only in the infrared to terahertz range,^31–33^ due to the confinement of SPE continuum. To date, very few plasmons with the frequency in the visible range have been predicted in 2D materials.^21^

Here, we investigated the plasmon excitation spectrum in a 2D DNL semimetal by using a tight-binding model in combination with first-principles calculations. We demonstrated that the 2D DNL states in the Lieb lattice can lead to anomalous plasmons with a stable frequency independent of the carrier density *n*, in sharp contrast to normal plasmons in 2D materials, such as graphene.^2,18,19^ Moreover, anomalous 2D plasmons are Landau-damping free in a wide-range of wave vectors, and have a high frequency, and high subwavelength confinement. We proposed a simple two-band model to interpret the density-independent frequency of plasmons. More interestingly, the SPE continuum can be effectively regulated by controlling the carrier density, to yield long-lived plasmons. Using first-principles calculations, we proposed a candidate material, 2D Be_2_C monolayer,^34^ to realize these interesting plasmons. Our calculations showed that the plasmons in the Be_2_C monolayer can survive up to the visible frequency region and propagate to large momentum transfer that has rarely been reported. Additionally, acoustic plasmon (AP) modes, characterized by linear dispersion in small momentum transfer, emerge in the electron-doped Be_2_C monolayer. The intensity and momentum transfer of these AP modes are much higher than those predicted in doped graphene^19,35^ and other 2D materials^36,37^ and thus likely detectable in experiments. Anomalous plasmons in the Lieb lattice hold great promise for the design of plasmonic devices.

## Methods and computational details

### A. Random-phase approximation

Under the random-phase approximation (RPA) approach, the polarization function reads^38,39^1
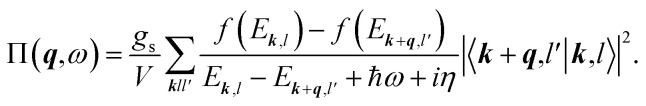
here *g*_s_ = 2 is the spin degeneracy, *V* is the area of the two-dimensional system, the broadening parameter is taken to be *η*, and the Fermi distribution function denoted by *f*(*E*) acts as a step function at *T* = 0. *E*_***k****l*_ and |***k***,*l*〉 are respectively the eigenvalues and eigenstates of the Hamiltonian matrix, where *l* represents the band indice. The dielectric function *ε*(***q***,*ω*) was determined from the polarization function using the equation:

2 *ε*(***q***,*ω*) = 1 − *V*_q_Π(***q***,*ω*),

where *V*_q_ is the Fourier transform of the Coulomb potential and can be written for a 2D system as3
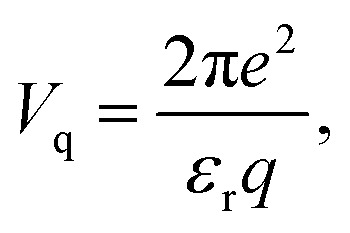
Notably, *V*_q_ is highly dependent on the dimensionality of the materials and becomes *V*_q_ = 4π*e*^2^/(*ε*_r_*q*^2^) for 3D materials, and 
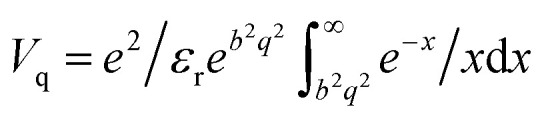
 for 1D materials,^40^ where *ε*_r_ is the background dielectric constant, and *b* is the width of the 1D materials. The dynamical loss function, which is related to the momentum-resolved electron energy loss spectrum (EELS), can be expressed as

4 *ε*_loss_(***q***,*ω*) = −Im[1/*ε*(***q***,*ω*)].

The plasmon is characterized by the peak of the dynamical loss function.

### B. First-principles calculations

Our first-principles calculations were performed using density functional theory, as implemented in the Vienna *ab initio* simulation package (VASP)^41^ and GPAW codes,^42^ which both employ the projector augmented-wave method^43^ to model interactions between electrons and ions with an energy cutoff of 500 eV. The generalized gradient approximation^44^ in the form of Perdew–Burke–Ernzerhof^45^ was adopted for the exchange–correlation functional. A vacuum space of 20 Å was used along the *z*-direction to exclude the interaction between neighbouring images. Structure relaxation and electronic properties were calculated using VASP on the 13 × 13 × 1 *k*-point mesh. The lattice constants and the atomic positions were fully relaxed until the atomic forces on the atoms were less than 0.01 eV Å^−1^ and the total energy change was less than 10^−5^ eV. The electron doping effect was simulated by adding or extracting extra electrons to the lattices in a homogeneous background charge of opposite sign. The band structures on the basis of maximally localized Wannier functions (MLWFs) were determined using WANNIER90 package.^46^

The dynamic dielectric function and loss function were performed using linear response calculations^47^ as implemented in the GPAW code. A denser *k*-point grid of 61 × 61 × 1 was used to include and accurate description of intra-band transitions. The dielectric matrix for in-plane wave vector **q** was calculated in random phase approximation (RPA) as:^47^5
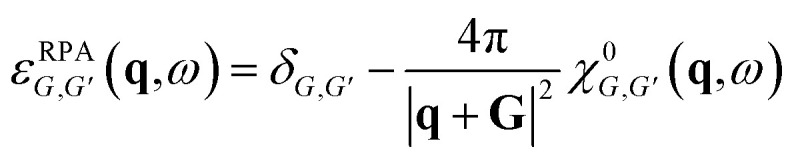


We consider up to 40 empty bands to describe the response function. A cut-off of 50 eV for reciprocal lattice vectors and a broadening parameter *η* = 0.05 eV were used to account for local field effects. In order to avoid the interaction between the periodic replicas, a 2D truncated Coulomb kernel was taken.^48^

## Results and discussion

### A. Two-band model of a 2D DNL

**Fig. 1:**
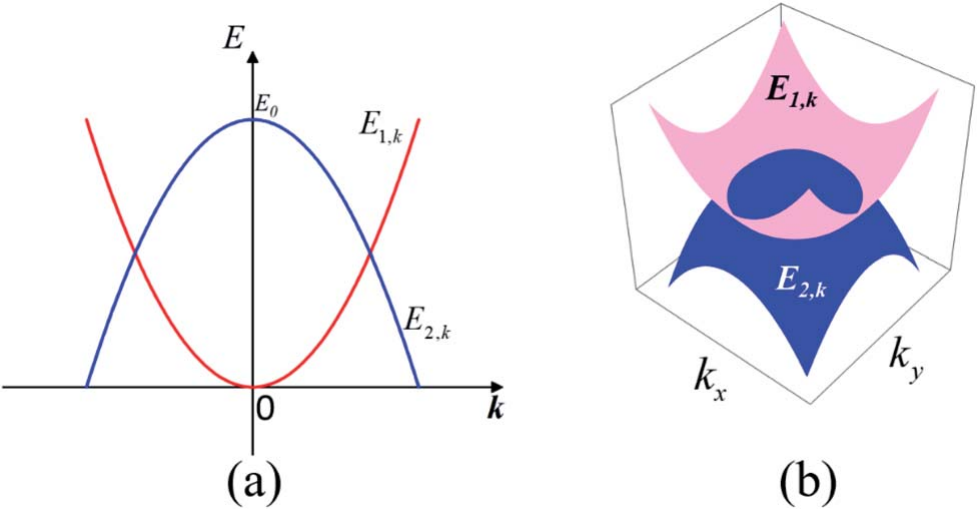
(a) Schematic representation of the two-band model of the 2D DNL formed from the cross of two parabolic bands. (b) The profiles of the 2D DNL in the reciprocal space.

We started from a simple model for the DNL in 2D materials to demonstrate the possibility of carrier-density-independent plasmons. We supposed that the DNL is formed from two crossing bands described by parabolic dispersion relations as follows:6
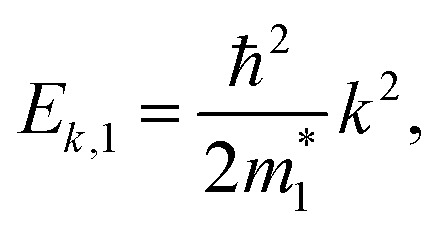
7
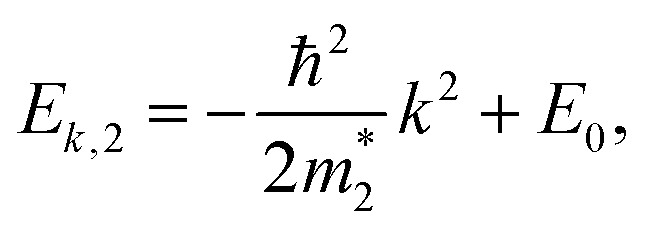
with the effective masses 
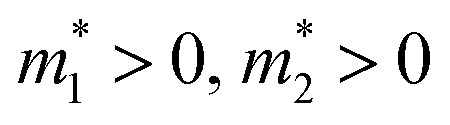
 and *E*_0_ > 0, as shown in Fig. 1. Assuming that the electron wavefunctions of the two bands are orthogonal (*e.g.* they arise from p_*z*_ and p_*xy*_ orbitals, respectively), the inter-band transition of electrons is prohibited.

The intra-band contribution of band *n* to the polarization functions reads:8
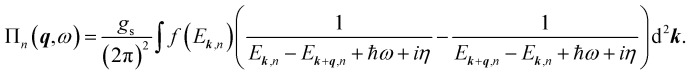


Under the long-wavelength limit, it can be reduced to:9
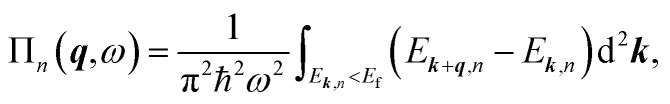
as the Fermi level *E*_f_ varies from 0 to *E*_0_. From eqn (9), we got the polarization function (see Sec. S2 of the ESI for more details†):10
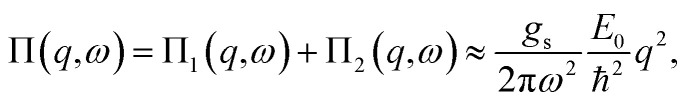
and the dispersion of the plasmon mode of this system:11
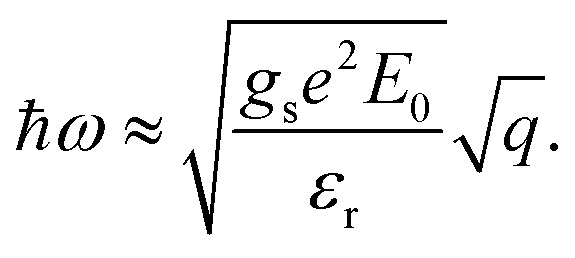
Notably, the plasmon spectrum is independent of the Fermi level (carrier density) and the effect masses of electrons.

### B. Tight-binding model for 2D plasmons in a Lieb lattice

**Fig. 2:**
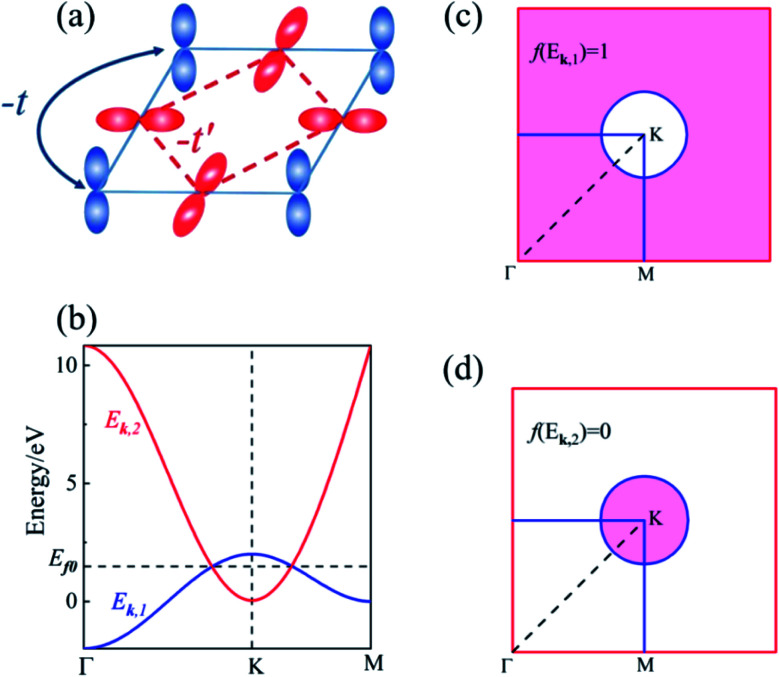
Tight-binding model of the 2D p-orbital Lieb lattice with a Dirac nodal-line electronic structure. (a) Lieb lattice of p_*z*_ and p_*x*,*y*_ orbitals. (b) The lower two bands of the TB model, *E*_***k***,1_ and *E*_***k***,2_, with *t* = 0.5 eV, *t*′ = 2.7 eV, *Δ* = 10.84 eV. (c and d) The Brillouin zone of the Lieb lattice. The shaded areas in (c) and (d) represent the momentum regions of *E*_***k***,1_ < *E*_f0_ and *E*_***k***,2_ < *E*_f0_, respectively.

We then considered the DNL in the 2D Lieb lattice proposed in our previous work,^34^ as shown in Fig. 2(a). The Lieb lattice is composed of two square sublattices of p_*z*_ and p_*x*,*y*_ orbitals, respectively. Electron hopping between adjacent sites is prohibited due to the orthogonal feature between p_*z*_ and p_*x*,*y*_ orbitals. Therefore, only electron hopping within the sublattices characterized by −*t* and −*t*′ (*t* > 0, *t*′ > 0) was taken into account in our tight-binding (TB) Hamiltonian:12
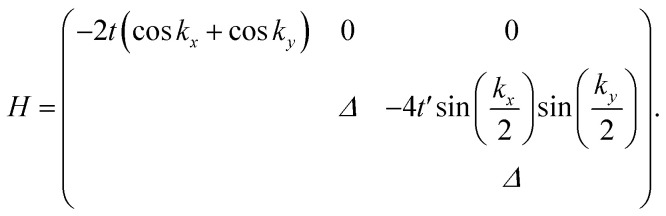
Here, *Δ* represents the on-site energy difference between two sublattices. Spin–orbit coupling (SOC) was not taken in account.

The lower two bands, *E*_***k***,1_ and *E*_***k***,2_ of the TB Hamiltonian,

13 *E*_***k***,1_ = −2*t*(cos *k*_*x*_ + cos *k*_*y*_),

14
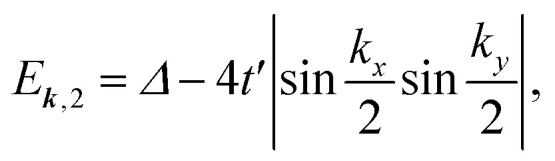
cross and form a Dirac nodal-line (DNL) centered at the *K* point, as (*t* + *t*′) > *Δ*/4. Assuming that there are two residual electrons per unit cell, the Fermi level *E*_f0_ is right at the band touching points, as shown in Fig. 2(b). The eigenvectors of the two bands are contributed respectively from the p_*z*_ and p_*x*,*y*_ orbitals and thus orthogonal to each other. The inter-band excitation of electrons between the two bands is forbidden and thus the contribution of inter-band transition to the polarization function can be omitted, similar to the case of the above two-band model.

In the long-wavelength limit, the polarization function can be evaluated as follows:15
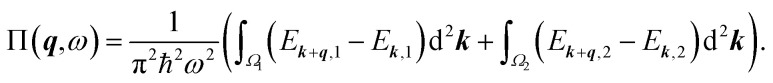
Here *Ω*_1_ and *Ω*_2_ represent the momentum regions of *E*_***k***,1_ < *E*_f_ and *E*_***k***,2_ < *E*_f_, as shown by the shaded areas in Fig. 2(c and d), respectively. The plasmon dispersion identified as the roots of *ε*(***q***,*ω*) = 0 can be reduced approximated to16
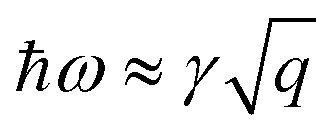
with 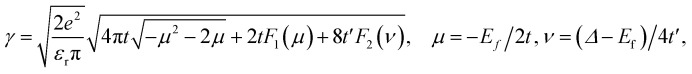 and 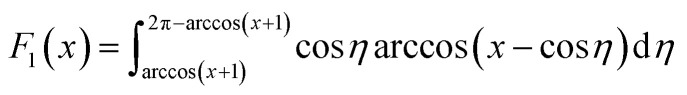, 
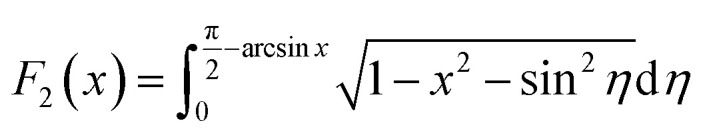
 (see Sec. S3 of the ESI for more details†). The plasmon modes are isotropic and satisfies *ω* ∝ *q*^1/2^ in the long-wavelength limit, similar to that of 2D electron gas.^28^

**Fig. 3:**
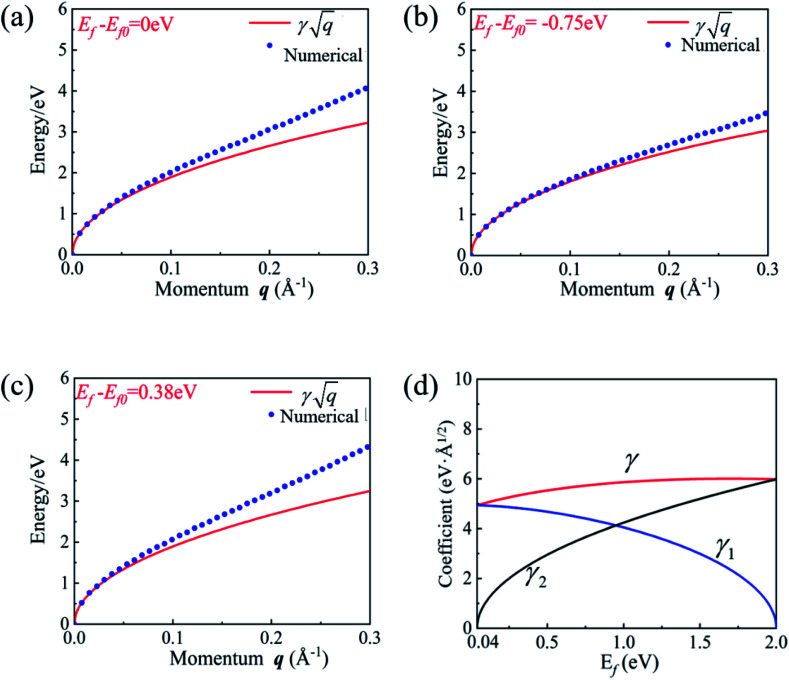
(a–c) The energy spectra of plasmons along the *Γ*–*M* direction for three different Fermi levels obtained by eqn (16) (solid red lines) and numerical calculations of eqn (8) without the long-wavelength limit (blue dots). (d) The relationship between the coefficient *γ*(*γ*_1_,*γ*_2_) and Fermi level *E*_f_ as the Fermi level was pushed from the bottom of *E*_***k***,2_ (0.04 eV) to the top of *E*_***k***,1_ (2.0 eV). The TB parameters are listed in Sec. S4 of the ESI.†

We also calculated the plasmon spectra using eqn (8)*via* numerical integration without long-wavelength approximation. The plasmon spectra along the *Γ*–*M* direction of the Lieb lattice with different Fermi energy (*E*_f_) are plotted in Fig. 3(a–c). Obviously, the data obtained directly from eqn (16) agree well with those from numerical calculations in the long wavelength region. Notably, both *μ* and *ν* are dependent on the Fermi level *E*_f_. The dependence of *γ* on the Fermi level was plotted in Fig. 3(d). As the Fermi level is pushed from the bottom of *E*_***k***,2_ (0.04 eV) to the top of *E*_***k***,1_ (2.0 eV), *γ* changes slightly from 4.93 to 6.0 eV Å^1/2^. This scenario differs significantly from that of traditional 2D systems,^18,28,29^ whose frequency strongly depends on carrier density, and reflects the unique characteristics of the 2D DNL. This TB model is also consistent with the two-band model, in which the energy difference between the bottom of *E*_***k***,2_ and the top of *E*_***k***,1_ is *E*_0_ = 1.96 eV. According to eqn (11), we have *γ* = 6.13 eV Å^1/2^, which is very close to the values (4.93–6.0 eV Å^1/2^) obtained from the TB model.

In order to reveal the relation between *γ* and *E*_f_, we divided *γ* as *γ*^2^ = *γ*_1_^2^ + *γ*_2_^2^, with 
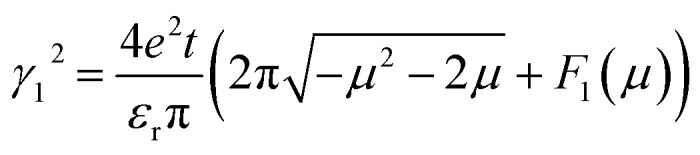
 and 
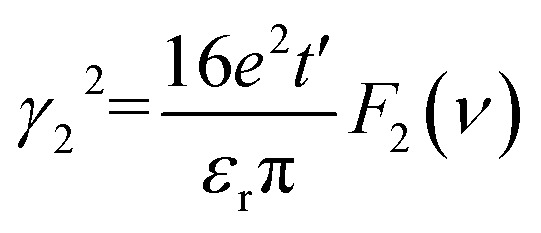
. Here, *γ*_1_ and *γ*_2_ represent the contributions of *E*_***k***,1_ and *E*_**k**,2_, respectively. The variation of *γ*_1_ and *γ*_2_ as a function of Fermi energy is plotted in Fig. 3(d). As *E*_f_ increases from the bottom of the band *E*_***k***,2_ to the top of the band *E*_***k***,1_, *γ*_1_ gradually decreases and finally becomes zero when *E*_f_ reaches the top of the band *E*_***k***,1_, while *γ*_2_ shows an opposite trend. As a result, *γ* becomes insensitive to the Fermi energy (or carrier density).

### C. 2D plasmons in the Be_2_C monolayer

**Fig. 4:**
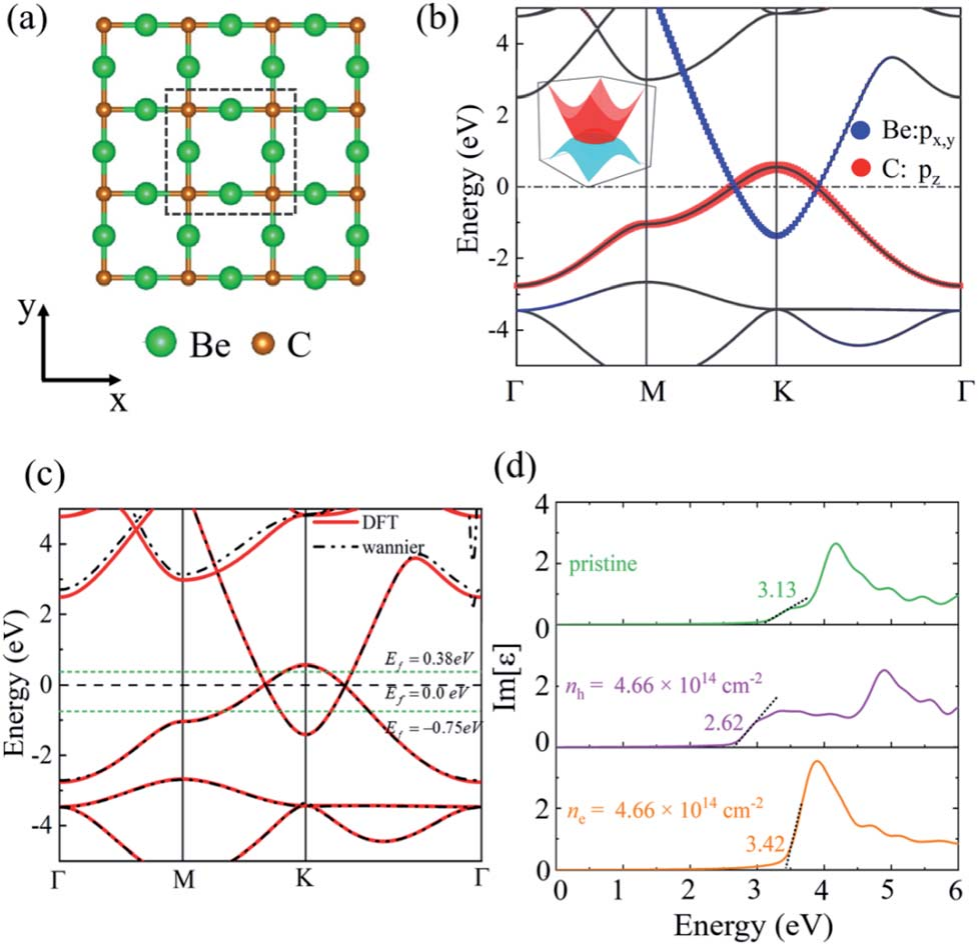
(a) The Lieb lattice of the Be_2_C monolayer. (b) The orbital-resolved band structure of the Be_2_C monolayer. The 3D band structure near the *K* point is shown in the inset. (c) Comparison of the band structure obtained from DFT methods (solid red lines) and MLWFs (dash-dotted black lines). Green dotted lines show the position of the Fermi level at different doping levels. (d) Imaginary part of the dielectric function of the pristine and doped Be_2_C monolayer. Black dotted lines show an approximate linear fit used to estimate the threshold energy of inter-band transitions.

To achieve anomalous plasmons in realistic materials, we considered a 2D Be_2_C monolayer proposed in our previous work.^34^ The Be_2_C monolayer possesses a line-centered square (Lieb) lattice with a lattice constant of 3.276 Å, where Be and C reside at the line-center and vertex sites, respectively, as shown in Fig. 4(a). The stability and plausibility of the Be_2_C monolayer have been verified from first-principles calculations.^34^ From the orbital-resolved electronic band structure plotted in Fig. 4(b), one can see clearly that the two bands cross along the *M*–*K* and *Γ*–*K* directions right at the Fermi level, forming a nodal ring centered at the *K* point. The two bands near the Fermi level are contributed mainly by the p_*x*,*y*_ orbitals of Be atoms and the p_*z*_ orbital of C atoms, respectively, meeting the requirement of the above models. Therefore, the 2D Be_2_C monolayer may serve as a promising candidate material to realize anomalous plasmons predicted by the two-band model and TB model.

We also plotted the band structure of the Be_2_C monolayer on the basis of maximally localized Wannier functions (MLWFs), which matches well with the DFT results in the energy range of [−5, 2] eV, as shown in Fig. 4(c). To mimic the effect of regulating the Fermi level, we doped holes (or electrons) into the Be_2_C monolayer with a concentration of 4.66 × 10^14^ cm^−2^. Compared with pristine Be_2_C, the Fermi level is pushed downward (or upward) by 0.75 eV (or 0.38 eV). The Dirac nodal-line states are still robust against doping (see Fig. S2 of the ESI†).

Notably, the two bands that form the DNL states near the Fermi level originate from different atomic orbitals: the p_*x*,*y*_ orbitals of Be atoms and the p_*z*_ orbital of C atoms. Electron excitation between the two bands (inter-band excitation) is not allowed due to the different symmetries of the two orbitals. This feature can be verified from the imaginary part of the dielectric function (Im *ε*) which describes the optical adsorption spectra of materials. Our first-principles calculations showed that the adsorption spectrum of pristine of Be_2_C has a threshold energy of about 3.13 eV, below which the inter-band excitation of electrons is prohibited, as shown in Fig. 4(d). For the hole- (or electron-) doped Be_2_C monolayer, the threshold energy becomes 2.62 eV (or 3.42 eV) at the doping concentration of 4.66 × 10^14^ cm^−2^. The threshold energy in the optical adsorption spectra of the DNL semimetallic Be_2_C monolayer is consistent with the theoretical models proposed in the previous sections and responsible for the anomalous plasmons.

**Fig. 5:**
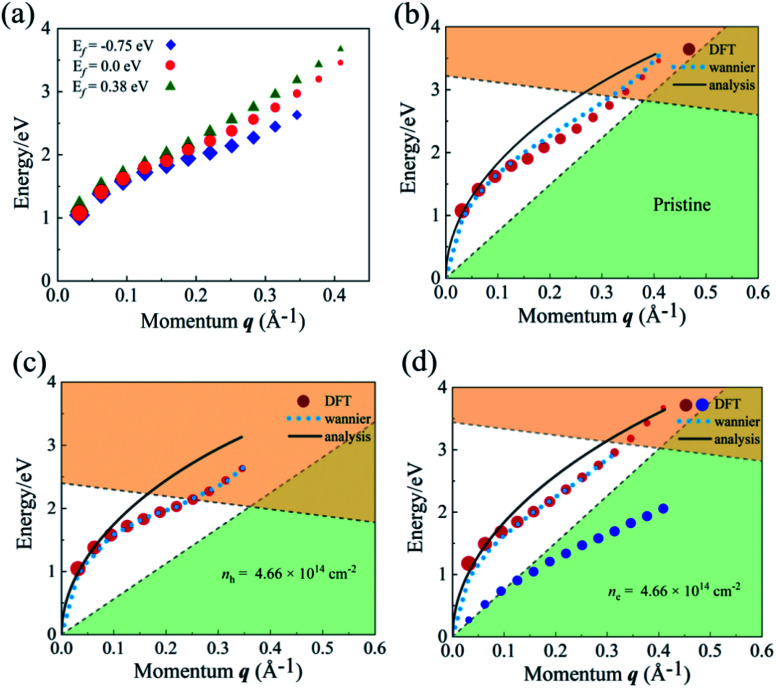
(a) Comparation of plasmon energy at different doping. Plasmon dispersion of the (b) pristine, (c) hole-doped and (d) electron-doped Be_2_C monolayer along the *Γ*–*M* direction. Red circles are results from DFT methods and the diameter of the circles is proportional to ln{Im[*ε*]}, indicating the strength of the plasmon mode. The results from MLWFs (blue dots) and analytical 
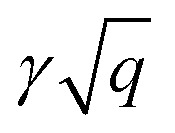
 dispersion (solid black lines) are also shown for comparison. The shaded areas indicate the SPE regions due to inter-band (yellow) and intra-band (green) transitions, respectively.

The plasmon dispersion curves of the Be_2_C monolayer along the *Γ*–*M* direction in the energy range of 0–4 eV are shown in Fig. 5, which were extracted from the peaks of EELS. The plasmon mode indicated by the red circles was named Dirac Plasmon (DP), because it originates from the collective electronic excitations of the Dirac nodal-line. For the small momentum transfer up to around 0.06 Å^−1^, the plasmon spectrum of the Be_2_C monolayer obtained from DFT calculations is in good agreement with the results of the TB model, which follows the universal long wavelength 
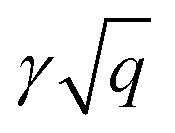
 behavior of 2D electron gas.^49^ But for large momentum transfer *q*, it deviates gradually from the 
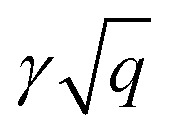
 rule. We ascribed it to the origins of the plasmons in the Be_2_C monolayer. In the small momentum region, the plasmons are mainly dominated by the intra-band transition of the two DNL bands. With the increase of momentum, however, the inter-band transition comes to occur and contributes to the polarization function. The consequent screening effect results in the redshift of the plasmon energy and deviation from the TB model.^22^ From the plasmon spectra under these three different doping conditions in Fig. 5(a), we can see that *γ* is nearly insensitive to the Fermi level. For further verification, we fitted the plasmon spectra of the Be_2_C monolayers in the low energy region by eqn (16) and got *γ* = 5.63 Å^1/2^ and 5.74 eV Å^1/2^ for the hole- and electron-doped Be_2_C monolayers with a concentration of 4.66 × 10^14^ cm^−2^, respectively, which are very close to that of the pristine Be_2_C monolayer, 5.76 eV Å^1/2^, in good agreement with the TB model.

To further include the contribution of other atomic orbitals, we also adopted a more complicated TB model by involving s- and p-orbitals in each site of the Lieb lattice to describe the electronic band structure and plasmonic properties of the Be_2_C monolayer. The parameters of the TB model can be obtained from the MLWF strategy by reproducing the band structure of the Be_2_C monolayer, as shown in Fig. 4(c). The plasmon modes calculated from the TB model agree well with the results of DFT calculations in the whole momentum region, as shown in Fig. 5(b–d).

Single-particle excitations (SPEs), due to intra-band and inter-band excitation (also known as Landau damping) dominate the plasmon damping processes. The SPE continuum is defined by the nonzero value of the imaginary part of the polarizability function, Im Π(***q***,*ω*).^18^ In our system, the borders of intra-band and inter-band SPE continuum can be determined approximately by ℏ^2^*q*^2^/2*m* + ℏ*v*_F_*q* and ℏ^2^*q*^2^/2*m* + ℏ*ω*_SPE_ − ℏ*v*_F2_*q*, respectively, in which ℏ^2^*q*^2^/2*m* is negligible compared with other terms as *q* → 0.^18,50,51^*v*_F_ represents the maximal electron velocity of the band across the Fermi level, which was set to 1.13 × 10^6^ m s^−1^, 0.85 × 10^6^ m s^−1^ and 1.14 × 10^6^ m s^−1^ for the pristine, hole-doped and electron-doped Be_2_C monolayer, respectively, according to our DFT calculations. *v*_F2_ is the electron velocity of the nearly flat band at around −3.5 eV in Fig. 4(c), which is responsible for the start of inter-band excitation. Our calculations showed that *v*_F2_ is insensitive to electron- or hole-doping and the maximum value of *v*_F2_ is about 1.9 × 10^5^ m s^−1^. The inter-band threshold frequency *ω*_SPE_ was identified as 3.13 eV, 2.62 eV, and 3.42 eV for pristine, hole-doped and electron-doped Be_2_C, respectively. Remarkably, the carrier-density-dependent *ω*_SPE_ is quite crucial for regulating the SPE continuum to achieve high-frequency plasmons. The shaded regions in Fig. 5(b–d) depict the SPE regions. When the plasmon mode hits the SPE continuum, it becomes damped and decays into electron–hole pairs. For pristine Be_2_C in Fig. 5(b), the plasmons are undamped up to 2.88 eV (*q* < 0.33 Å^−1^). In the hole-doped Be_2_C monolayer, the SPE continuum moves downward, as shown in Fig. 5(c), and the maximal frequency of the undamped plasmons decreases to 2.14 eV (*q* < 0.25 Å^−1^). In contrast, in the electron-doped Be_2_C monolayer, the improved *ω*_SPE_ raises the boundary of the inter-band SPE region, making the plasmons extend up to 3.10 eV (*q* < 0.37 Å^−1^), as shown in Fig. 5(d). Notably, the 2D plasmons in the visible frequency range remain challenging. Anomalous plasmons predicted in the Be_2_C monolayer offer a promising approach to reach this goal.

Another interesting scenario worth noting is that, for the electron-doped Be_2_C monolayer, a new type of plasmon mode emerges, which has a linear dispersion and coincides with the intra-band SPE boundary in small momentum transfer, as indicated by blue circles in Fig. 5(d). A similar plasmon mode defined as acoustic plasmon (AP) has been predicted in doped-graphene,^19,35^ but the intensity is too low. For the AP mode in the Be_2_C monolayer, however, the intensity is greatly enhanced in the high *q* region, and even exceeds the intensity of the DP. Although the AP mode enters into the electron–hole excitation area at seemingly small *q* (∼0.1 Å^−1^), the high intensity and relatively larger momentum transfer than other 2D materials like doped-graphene make it detectable in experiments.

**Fig. 6:**
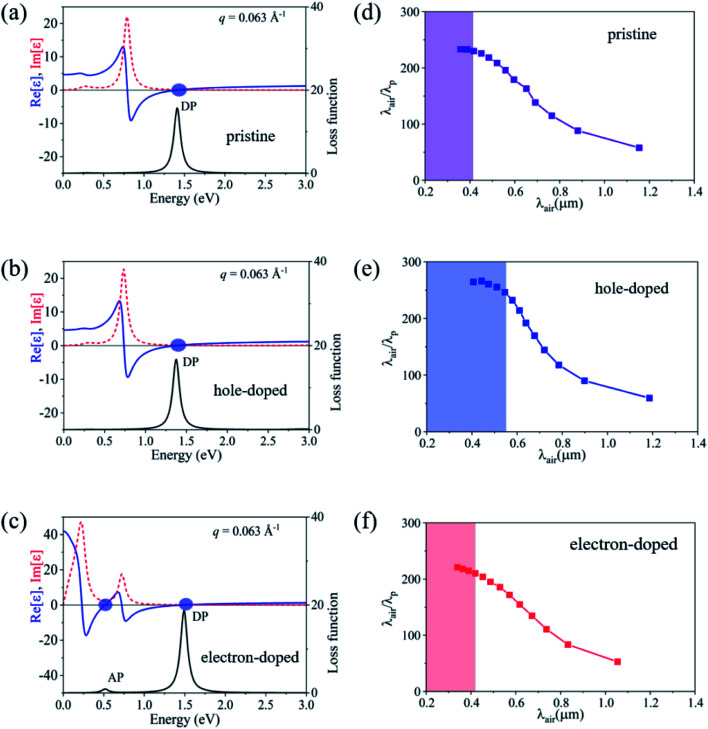
(a–c) Dielectric function and loss function of pristine, hole-doped and electron-doped Be_2_C monolayers at *q* = 0.063 Å^−1^. The upper panel is the real (blue solid lines) and imaginary part (red dashed lines) of the dielectric function, and the blue circles denote the zeros of Re*ε*(***q***,*ω*) corresponding to collective excitations. The lower panel is the corresponding loss function, in which the DP and AP modes are marked near the peaks. (d–f) Wave localization of the DP mode in pristine, hole-doped and electron-doped Be_2_C monolayers, respectively. The shaded areas correspond to SPE damping regions.

To verify the collective excitation features (rather than single-particle resonances) of the two-types of plasmon modes (DP and AP), we plotted the dielectric functions and loss functions of the Be_2_C monolayer at *q* = 0.063 Å^−1^ in Fig. 6. Collective excitation should fulfill Re*ε* = 0 with Im *ε*/*∂*_*ω*_Re*ε* > 0 at the energy where the peak of loss function occurs. If the Im *ε* presents a vanishing small value in the same energy region, the plasmon is undamped and lies out of the SPE regions.^25,52,53^ From Fig. 6 we can clearly see that, all conditions are fulfilled in the DP case, confirming the plasmonic nature of the DP mode. For the electron-doped Be_2_C monolayer, Im *ε* deviates slightly from zero at the energy where AP occurs. We attribute it to the artificial broadening effect of the two peaks in the Im *ε* spectrum in our DFT calculations.

We finally evaluate the wave localization (or wave shrinkage) of the DP in the Be_2_C monolayer from the confinement of the wavelength of a plasmon mode. Wave localization is qualified as *λ*_air_/*λ*_p_, in which *λ*_p_ = 2π/*q* is the wavelength of a plasmon wave and *λ*_air_ = 2π*c*/*ω* is the corresponding wavelength in air. The maximum wave localization of the undamped plasmons was determined to be 228, 231 and 213 for the pristine, hole-doped and electron-doped Be_2_C monolayer, respectively, as illustrated in Fig. 6(d–f). These values are larger than those predicted in doped-graphene (100–200)^54^ and bulked triangular 2D boron (∼150),^21^ demonstrating the subwavelength confinement of the plasmons in the Be_2_C monolayer which would be beneficial for miniaturization of plasmonic devices and savings of the operational power.^55^

We should mention that achieving visible-range plasmons in 2D materials remains challenging in experiments.^56^ For gate-doped graphene, plasmons can emerge in the terahertz to infrared range, which has been proved both theoretically and experimentally.^2,18,19,31–33^ Electron-doping was predicted to extend the frequency of 2D plasmons to the infrared region in MoS_2_ monolayers.^57^ Through electrochemically intercalating lithium into MoS_2_ nanoflakes, plasmon resonances in the visible and near UV wavelength ranges were realized in recent experiments.^58^ In present work, we predicted that the plasmon mode in the Be_2_C monolayer can extend to the visible range, even without doping, offering an alternative approach to achieve visible-range plasmons in 2D materials.

## Conclusions

In summary, we revealed anomalous plasmons inherited in the Lieb lattice with DNL electronic structures using a TB model in combination with first-principles calculations. Our study showed that the plasmons exhibit the unique features of a density-independent frequency, being Landau-damping free in a wide-range of wave vectors, a high frequency, and high subwavelength confinement, which can be attributed to the DNL states and symmetry-restricted inter-band excitation. The SPE continuum can be effectively regulated by controlling the carrier density, yielding long-lived plasmons. These interesting plasmonic properties can be realized in a 2D Be_2_C monolayer. More interestingly, acoustic plasmon (AP) modes, characterized by linear dispersion in small momentum transfer, emerge in the electron-doped Be_2_C monolayer. The intensity and momentum transfer of these AP modes is comparable to that of the DP modes and thus has high possibility to be detected in experiments. Our work is expected to offer a promising strategy for achieving stable plasmons in the visible light regime.

## Conflicts of interest

There are no conflicts to declare.

## Supplementary Material

NA-003-D0NA00759E-s001
