# Supplementary material for: Anomalous plasmons in a two-dimensional Dirac nodal-line Lieb lattice
Source: Nanoscale Adv. 2020 Dec 26;3(4):1127–35. doi: 10.1039/d0na00759e (PMC9419277; doi:10.1039/d0na00759e)
Supplement: NA-003-D0NA00759E-s001 [file NA-003-D0NA00759E-s001.pdf]

Electronic Supplementary Material (ESI) for Nanoscale Advances.
This journal is © The Royal Society of Chemistry 2020

Supplementary material for

Anomalous Plasmons in Two-Dimensional Dirac Nodal-Line Lieb Lattice

Chao Ding, Han Gao, Wenhui Geng, Mingwen Zhao*

School of Physics & State Key Laboratory of Crystal Materials, Shandong University, Jinan 250100, Shandong, China

S1. INTRA-BAND POLARIZATION FUNCTION

In this section, we will systematically derive the intra-band polarization function and its long-wavelength limit under the random-phase approximation (RPA) approach.

The intra-band polarization function of band $E_{\boldsymbol{k},n}$ is [1, 2]

$$\Pi_n(\boldsymbol{q},\omega)=\frac{g_s}{V}\sum_{\boldsymbol{k}}\frac{f(E_{\boldsymbol{k},n})-f(E_{\boldsymbol{k}+\boldsymbol{q},n})}{E_{\boldsymbol{k},n}-E_{\boldsymbol{k}+\boldsymbol{q},n}+\hbar\omega+i\eta}\left|\langle\boldsymbol{k}+\boldsymbol{q},n|\boldsymbol{k},n\rangle\right|^2. \quad (1)$$

We assume $\left|\langle\boldsymbol{k}+\boldsymbol{q},n|\boldsymbol{k},n\rangle\right|^2\approx 1$ in our discussion. Because of $E_{\boldsymbol{k},n}=E_{\boldsymbol{k}+\boldsymbol{G},n}$ ($\boldsymbol{G}$ is an arbitrary reciprocal lattice vector.), a standard replacement $\boldsymbol{k}\to-\boldsymbol{k}-\boldsymbol{q}$ can be performed in the term containing $f(E_{\boldsymbol{k}+\boldsymbol{q},n})$. This gives

$$\Pi_n(\boldsymbol{q},\omega)=\frac{g_s}{V}\sum_{\boldsymbol{k}}\left(\frac{f(E_{\boldsymbol{k},n})}{E_{\boldsymbol{k},n}-E_{\boldsymbol{k}+\boldsymbol{q},n}+\hbar\omega+i\eta}-\frac{f(E_{-\boldsymbol{k},n})}{E_{-\boldsymbol{k}-\boldsymbol{q},n}-E_{-\boldsymbol{k},n}+\hbar\omega+i\eta}\right). \quad (2)$$

Assuming that $E_{\boldsymbol{k},n}=E_{-\boldsymbol{k},n}$,

$$\Pi_n(\boldsymbol{q},\omega)=\frac{g_s}{V}\sum_{\boldsymbol{k}}\left(\frac{f(E_{\boldsymbol{k},n})}{E_{\boldsymbol{k},n}-E_{\boldsymbol{k}+\boldsymbol{q},n}+\hbar\omega+i\eta}-\frac{f(E_{\boldsymbol{k},n})}{E_{\boldsymbol{k}+\boldsymbol{q},n}-E_{\boldsymbol{k},n}+\hbar\omega+i\eta}\right). \quad (3)$$

Eq. (3) can also be written as an integral, this gives

$$\Pi_n(\boldsymbol{q},\omega)=\frac{g_s}{(2\pi)^2}\int\left(\frac{f(E_{\boldsymbol{k},n})}{E_{\boldsymbol{k},n}-E_{\boldsymbol{k}+\boldsymbol{q},n}+\hbar\omega+i\eta}-\frac{f(E_{\boldsymbol{k},n})}{E_{\boldsymbol{k}+\boldsymbol{q},n}-E_{\boldsymbol{k},n}+\hbar\omega+i\eta}\right)d^2\boldsymbol{k}. \quad (4)$$

Now, let $\eta\to 0$, we obtain

$$\Pi_n(\boldsymbol{q},\omega)=\frac{g_s}{(2\pi)^2}\int f(E_{\boldsymbol{k},n})\frac{2(E_{\boldsymbol{k}+\boldsymbol{q},n}-E_{\boldsymbol{k},n})}{\hbar^2\omega^2-(E_{\boldsymbol{k}+\boldsymbol{q},n}-E_{\boldsymbol{k},n})^2}d^2\boldsymbol{k}. \quad (5)$$

In the long-wavelength limit, where $\hbar\omega$? $\left|E_{\boldsymbol{k}+\boldsymbol{q},n}-E_{\boldsymbol{k},n}\right|$,

$$\Pi_n(\boldsymbol{q},\omega)=\frac{1}{\pi^2\hbar^2\omega^2}\int f(E_{\boldsymbol{k},n})(E_{\boldsymbol{k}+\boldsymbol{q},n}-E_{\boldsymbol{k},n})d^2\boldsymbol{k}. \quad (6)$$

S2. DETAILS OF THE ELECTRON-HOLE MODEL

We supposed that the two-dimensional (2D) DNL is formed from two crossing bands described by parabolic dispersion relations as follows:

$$E_{\boldsymbol{k},1} = \frac{\hbar^2}{2m_1^*}k^2, E_{\boldsymbol{k},2} = -\frac{\hbar^2}{2m_2^*}k^2 + E_0, \tag{7}$$

with $m_1^* > 0, m_2^* > 0$ and E_0 >0. Assuming that electron wavefunctions of the two bands are orthogonal, the inter-band transition of electrons is prohibited. We therefore only considered the intra-band contribution to the polarization functions.

Under long-wavelength limit, the polarization function of band $E_{\boldsymbol{k},1}$ is

$$\Pi_1(\boldsymbol{q},\omega) = \frac{1}{\pi^2\hbar^2\omega^2}\int_{E_{\boldsymbol{k},1}<E_f}(E_{\boldsymbol{k}+\boldsymbol{q},1} - E_{\boldsymbol{k},1})d^2\boldsymbol{k}. \tag{8}$$

Due to the region where $E_{\boldsymbol{k},1} < E_f$ has the symmetry of space inversion,

$$\Pi_1(\boldsymbol{q},\omega) = \frac{1}{2\pi^2\hbar^2\omega^2}\int_{E_{\boldsymbol{k},1}<E_f}(E_{\boldsymbol{k}+\boldsymbol{q},1} + E_{-\boldsymbol{k}+\boldsymbol{q},1} - E_{\boldsymbol{k},1} - E_{-\boldsymbol{k},1})d^2\boldsymbol{k}. \tag{9}$$

Considering that $E_{\boldsymbol{k},1} = E_{-\boldsymbol{k},1}$, Eq. (9) can also be written as

$$\Pi_1(\boldsymbol{q},\omega) = \frac{1}{2\pi^2\hbar^2\omega^2}\int_{E_{\boldsymbol{k},1}<E_f}(E_{\boldsymbol{k}+\boldsymbol{q},1} + E_{\boldsymbol{k}-\boldsymbol{q},1} - 2E_{\boldsymbol{k},1})d^2\boldsymbol{k}. \tag{10}$$

Considering the Taylor's expansion to the second order,

$$E_{\boldsymbol{k}+\boldsymbol{q},1} - E_{\boldsymbol{k},1} \approx \nabla_{\boldsymbol{k}}E_{\boldsymbol{k},1}\cdot\boldsymbol{q} + \frac{1}{2}(\boldsymbol{q}\cdot\nabla_{\boldsymbol{k}})^2E_{\boldsymbol{k},1}. \tag{11}$$

Combining Eq. (7), Eq. (10) and Eq. (11), we finally obtain

$$\Pi_1(q,\omega) = \frac{g_s}{4\pi\omega^2}\frac{q^2}{m_1^*}k_{1f}^2, \tag{12}$$

where k_{1f} is the Fermi wave vector of band $E_{\boldsymbol{k},1}$. The polarization function can also be written as $\Pi_1(q,\omega) = \frac{n_1 q^2}{m_1^*\omega^2}$, where $n_1 = \frac{g_s}{4\pi}k_{1f}^2$ is the carrier density of band $E_{\boldsymbol{k},1}$.

It is exactly the polarization function of the 2D electron gas [3].

The polarization function of band $E_{\boldsymbol{k},2}$ can be derived in a similar way. Under long-wavelength limit,

$$\Pi_2(\boldsymbol{q},\omega)=\frac{1}{\pi^2\hbar^2\omega^2}\int_{E_{\boldsymbol{k},2}<E_f}(E_{\boldsymbol{k}+\boldsymbol{q},2}-E_{\boldsymbol{k},2})d^2\boldsymbol{k}\ . \tag{13}$$

Assuming that band $E_{\boldsymbol{k},2}$ is periodic of reciprocal lattice vector, we obtain $\int_{\Omega'}(E_{\boldsymbol{k}+\boldsymbol{q},2}-E_{\boldsymbol{k},2})d^2\boldsymbol{k}=0$, where Ω' is the Brillouin zone. Hence,

$$\begin{aligned}\Pi_2(\boldsymbol{q},\omega)&=-\frac{1}{\pi^2\hbar^2\omega^2}\int_{E_{\boldsymbol{k},2}>E_f}(E_{\boldsymbol{k}+\boldsymbol{q},2}-E_{\boldsymbol{k},2})d^2\boldsymbol{k}\\&=-\frac{1}{2\pi^2\hbar^2\omega^2}\int_{E_{\boldsymbol{k},2}>E_f}(E_{\boldsymbol{k}+\boldsymbol{q},2}+E_{\boldsymbol{k}-\boldsymbol{q},2}-2E_{\boldsymbol{k},2})d^2\boldsymbol{k}\end{aligned} \tag{14}$$

Combining Eq. (7) and Eq. (14), and performing a second order Taylor's expansion similar to Eq. (11), we can obtain

$$\Pi_2(q,\omega)=\frac{g_s}{4\pi\omega^2}\frac{q^2}{m_2^*}k_{2f}^2\ , \tag{15}$$

where k_{2f} is the Fermi wave vector of band $E_{\boldsymbol{k},2}$. Then, we obtain the polarization function of this system

$$\Pi(q,\omega)=\Pi_1(q,\omega)+\Pi_2(q,\omega)=\frac{g_s}{4\pi\omega^2}(\frac{k_{1f}^2}{m_1^*}+\frac{k_{2f}^2}{m_2^*})q^2\ . \tag{16}$$

Considering that $\frac{\hbar^2}{2m_1^*}{k_{1f}}^2=-\frac{\hbar^2}{2m_2^*}{k_{2f}}^2+E_0=E_f$, we finally obtain

$$\Pi(q,\omega)=\frac{g_s}{2\pi\omega^2}\frac{E_0}{\hbar^2}q^2\ . \tag{17}$$

The dispersion of plasmon mode of this system can be written as

$$\hbar\omega=\sqrt{\frac{g_s e^2 E_0}{\varepsilon_r}}\sqrt{q}\ . \tag{18}$$

S3. DETAILS OF THE TIGHT-BINDING MODEL FOR 2D PLASMONS IN LIEB LATTICE

We first derive the intra-band polarization function of $E_{\boldsymbol{k},1}$.

The dispersion of band $E_{\boldsymbol{k},1}$ is

$$E_{\boldsymbol{k}1} = -2t(\cos k_x + \cos k_y). \tag{19}$$

Under long-wavelength limit, the polarization function $\Pi_1(\boldsymbol{q},\omega)$ is

$$\Pi_1(\boldsymbol{q},\omega) = \frac{1}{\pi^2 \hbar^2 \omega^2} \int_{\Omega_1} (E_{\boldsymbol{k}+\boldsymbol{q},1} - E_{\boldsymbol{k}1}) d^2\boldsymbol{k}, \tag{20}$$

where Ω_1: $\cos(k_x) + \cos(k_y) > \mu (\mu = -\frac{E_f}{2t})$, is the region where $E_{\boldsymbol{k},1} < E_f$, as shown by shaded areas in Fig. 2(c). Assuming the whole Brillouin zone is Ω, and Ω_1' is the region shown by white in Fig. 2(c) , hence

$$\int_{\Omega_1} (E_{\boldsymbol{k}+\boldsymbol{q},1} - E_{\boldsymbol{k},1}) d^2\boldsymbol{k} = \int_{\Omega} (E_{\boldsymbol{k}+\boldsymbol{q},1} - E_{\boldsymbol{k},1}) d^2\boldsymbol{k} - \int_{\Omega_1'} (E_{\boldsymbol{k}+\boldsymbol{q},1} - E_{\boldsymbol{k},1}) d^2\boldsymbol{k}. \tag{21}$$

Considering that the band $E_{\boldsymbol{k},1}$ is periodic of reciprocal lattice vector, we can obtain $\int_{\Omega} (E_{\boldsymbol{k}+\boldsymbol{q},1} - E_{\boldsymbol{k},1}) d^2\boldsymbol{k} = 0$, hence

$$\int_{\Omega_1} (E_{\boldsymbol{k}+\boldsymbol{q},1} - E_{\boldsymbol{k},1}) d^2\boldsymbol{k} = -\int_{\Omega_1'} (E_{\boldsymbol{k}+\boldsymbol{q},1} - E_{\boldsymbol{k},1}) d^2\boldsymbol{k}. \tag{22}$$

The region Ω_1' is bounded by $\cos(k_x) + \cos(k_y) = \mu$, which contains

$$\begin{aligned} k_{x1} &= \arccos(\mu + 1), \\ k_{x2} &= 2\pi - \arccos(\mu + 1), \\ k_{y1} &= \arccos(\mu - \cos k_x), \\ k_{y2} &= 2\pi - \arccos(\mu - \cos k_x), \end{aligned} \tag{23}$$

with $\mu < 0$. Substituting Eq. (19) into Eq. (20) and using the relationship of Eq. (22), we get

$$\Pi_1(\boldsymbol{q},\omega) = -\frac{2t}{\pi^2\hbar^2\omega^2}\Big(\int_{\Omega_1'}[(1-\cos q_x)\cos k_x + (1-\cos q_y)\cos k_y]d^2\boldsymbol{k} + \int_{\Omega_1'}(\sin k_x \sin q_x + \sin k_y \sin q_y)d^2\boldsymbol{k}\Big). \tag{24}$$

To calculate Eq. (24), the following calculation was performed:

$$\begin{aligned}
&\int_{\Omega_1'}\cos k_x d^2\boldsymbol{k} = \int_{k_{x1}}^{k_{x2}} dk_x \int_{k_{y1}}^{k_{y2}}\cos k_x dk_y = -4\pi\sqrt{-\mu^2-2\mu} - 2F_1(\mu),\\
&\int_{\Omega_1'}\cos k_y d^2\boldsymbol{k} = \int_{\Omega_1'}\cos k_x d^2\boldsymbol{k},\\
&\int_{\Omega_1'}\sin k_x d^2\boldsymbol{k} = \int_{\Omega_1'}\sin k_y d^2\boldsymbol{k} = 0,
\end{aligned} \tag{25}$$

where $F_1(x)$ is a function defined as

$$F_1(x) = \int_{\arccos(x+1)}^{2\pi-\arccos(x+1)}\cos\eta \arccos(x-\cos\eta)d\eta\ . \tag{26}$$

Substituting Eq. (25) into Eq. (24), we finally obtain

$$\Pi_1(\boldsymbol{q},\omega) = \frac{1}{\pi^2\hbar^2\omega^2}\alpha(2-\cos q_x - \cos q_y)\,, \tag{27}$$

with $\alpha{=}8\pi t\sqrt{-\mu^2-2\mu} + 4tF_1(\mu)$.

The intra-band polarization function of $E_{\boldsymbol{k},2}$ can be derived in a similar way.

The dispersion of band $E_{\boldsymbol{k},2}$ is

$$E_{\boldsymbol{k},2} = \Delta - 4t'\sin\frac{k_x}{2}\sin\frac{k_y}{2}\,. \tag{28}$$

And the polarization function $\Pi_2(\boldsymbol{q},\omega)$ under long-wavelength limit is

$$\Pi_2(\boldsymbol{q},\omega) = \frac{1}{\pi^2\hbar^2\omega^2}\int_{\Omega_2}(E_{\boldsymbol{k}+\boldsymbol{q},2} - E_{\boldsymbol{k},2})d^2\boldsymbol{k}\,, \tag{29}$$

here Ω_2: $\sin(\frac{k_x}{2})\sin(\frac{k_y}{2}) > \nu(\nu{=}\frac{\Delta - E_f}{4t'})$, is the region where $E_{\boldsymbol{k},2} < E_f$, as shown by shaded areas in Fig. 2(d). The region Ω_2 is bounded by

$$
\begin{aligned}
k_{x1} &= 2\arcsin\nu, \\
k_{x2} &= 2\pi - 2\arcsin\nu, \\
k_{y1} &= 2\arcsin(\frac{\nu}{\sin(\frac{k_x}{2})}), \\
k_{y2} &= 2\pi - 2\arcsin(\frac{\nu}{\sin(\frac{k_x}{2})}).
\end{aligned} \tag{30}
$$

Substituting Eq. (28) into Eq. (29), then we get

$$
\begin{aligned}
\Pi_2(q,\omega) = \frac{-4t'}{\pi^2\hbar^2\omega^2}(&\int_{\Omega_2}(\cos\frac{q_x}{2}\cos\frac{q_y}{2}-1)\sin\frac{k_x}{2}\sin\frac{k_y}{2}d^2\boldsymbol{k} \\
&+\int_{\Omega_2}\sin\frac{k_x}{2}\cos\frac{k_y}{2}\cos\frac{q_x}{2}\sin\frac{q_y}{2}d^2\boldsymbol{k} \\
&+\int_{\Omega_2}\cos\frac{k_x}{2}\sin\frac{k_y}{2}\sin\frac{q_x}{2}\cos\frac{q_y}{2}d^2\boldsymbol{k} \\
&+\int_{\Omega_2}\cos\frac{k_x}{2}\cos\frac{k_y}{2}\sin\frac{q_x}{2}\sin\frac{q_y}{2}d^2\boldsymbol{k}).
\end{aligned} \tag{31}
$$

To calculate Eq. (31), the following calculation was performed:

$$
\begin{aligned}
&\int_{\Omega_2}\sin\frac{k_x}{2}\sin\frac{k_y}{2}d^2\boldsymbol{k} = \int_{k_{x1}}^{k_{x2}}dk_x\int_{k_{y1}}^{k_{y2}}\sin\frac{k_x}{2}\sin\frac{k_y}{2}dk_y = 16F_2(\nu), \\
&\int_{\Omega_2}\sin\frac{k_x}{2}\cos\frac{k_y}{2}d^2\boldsymbol{k} = \int_{\Omega_2}\cos\frac{k_x}{2}\sin\frac{k_y}{2}d^2\boldsymbol{k} = 0, \\
&\int_{\Omega_2}\cos\frac{k_x}{2}\cos\frac{k_y}{2}d^2\boldsymbol{k} = 0,
\end{aligned} \tag{32}
$$

where $F_2(x)$ is a function defined as

$$
F_2(x) = \int_0^{\frac{\pi}{2}-\arcsin x}\sqrt{1-x^2-\sin^2\eta}\,d\eta\,. \tag{33}
$$

Substituting Eq. (32) into Eq. (31), we finally obtain

$$
\Pi_2(\boldsymbol{q},\omega) = \frac{1}{\pi^2\hbar^2\omega^2}\beta(1-\cos\frac{q_x}{2}\cos\frac{q_y}{2}), \tag{34}
$$

with $\beta = 64t'F_2(\nu)$.

Then the polarization function of this system can be written as

$$\Pi(\boldsymbol{q},\omega)=\Pi_1(\boldsymbol{q},\omega)+\Pi_2(\boldsymbol{q},\omega)$$
$$=\frac{1}{\pi^2\hbar^2\omega^2}[\alpha(2-\cos q_x-\cos q_y)+\beta(1-\cos\frac{q_x}{2}\cos\frac{q_y}{2})]. \tag{35}$$

The plasmon dispersion identified as the roots of $\varepsilon(\boldsymbol{q},\omega)=0$ is

$$\hbar\omega=\sqrt{\frac{2e^2}{\varepsilon_r q\pi}}\sqrt{\alpha\left(2-\cos q_x+\cos q_y\right)+\beta\left(1-\cos\frac{q_x}{2}\cos\frac{q_y}{2}\right)}. \tag{36}$$

Making the substitution $\cos x\to 1-\frac{1}{2}x^2$, Eq. (36) can be reduced to:

$$\hbar\omega\approx\gamma\sqrt{q}\,. \tag{37}$$

with $\gamma=\sqrt{\frac{2e^2}{\varepsilon_r\pi}}\sqrt{4\pi t\sqrt{-\mu^2-2\mu}+2tF_1(\mu)+8t'F_2(\nu)}$.

S4. SOME COMPUTATIONAL DETAILS OF TIGHT-BINDING MODEL

The parameters we use to calculate in Sec. Ⅲ(B) are $t=0.5eV, t'=2.7eV, \Delta=10.84eV$, $\varepsilon_r=1.5$ $a=3.278\,\mathring{A}$ $\eta=0.05eV$. The tight-binding parameters come from fitting the bands of our TB model to Be_2C monolayer, as shown in Fig. S1. ε_r that we use to calculate is derived from fitting the plasmon dispersion of the more complicated TB model constructed by WANNIER90 [4] to the results of GPAW [5]. a is the lattice constant of Be_2C monolayer.

S5. ELECTRONIC BAND STRUCTURES OF DIFFERENT DOPPING LEVELS

The electronic band structures of pristine, hole-doped and electron-doped Be_2C monolayer are shown in Fig. S2.

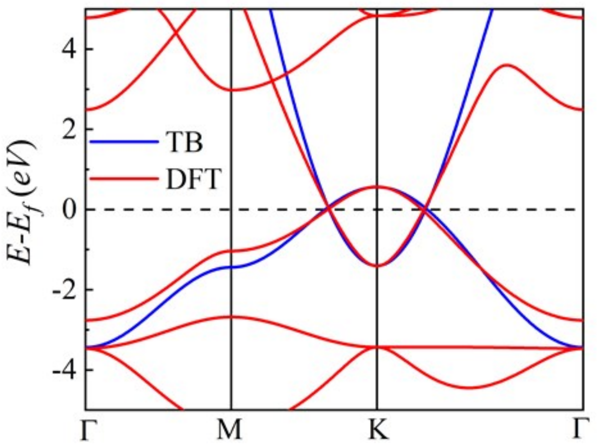

Figure S1. Band structure obtained from TB model and density-functional theory calculations of Be_2C monolayer.

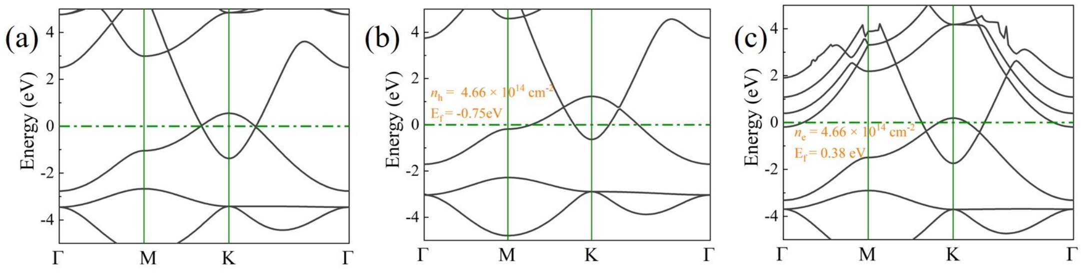

Figure S2. The electronic band structures of (a) pristine, (b) hole-doped and (c) electron-doped Be_2C monolayer, respectively.

[1] N. Wiser, Dielectric Constant with Local Field Effects Included, Phys. Rev. **129**, 62 (1963).

[2] S.L. Adler, Quantum Theory of the Dielectric Constant in Real Solids, Phys. Rev. **126**, 413 (1962).

[3] F. Stern, Polarizability of a Two-Dimensional Electron Gas, Phys. Rev. Lett. **18,** 546 (1967).

[4] A.A. Mostofi, J.R. Yates, Y.-S. Lee, I. Souza, D. Vanderbilt and N. Marzari, wannier90: A tool for obtaining maximally-localised Wannier functions, Comput. Phys. Commun. **178**, 685 (2008).

[5] J. Enkovaara, C. Rostgaard, J.J. Mortensen, J. Chen, M. Dułak, L. Ferrighi, J. Gavnholt, C. Glinsvad, V. Haikola, H.A. Hansen, H.H. Kristoffersen, M. Kuisma, A.H. Larsen, L. Lehtovaara, M. Ljungberg, O. Lopez-Acevedo, P.G. Moses, J. Ojanen, T. Olsen, V. Petzold, N.A. Romero, J. Stausholm-Møller, M. Strange, G.A. Tritsaris, M. Vanin, M. Walter, B. Hammer, H. Häkkinen, G.K.H. Madsen, R.M. Nieminen, J.K. Nørskov, M. Puska, T.T. Rantala, J. Schiøtz, K.S. Thygesen and K.W. Jacobsen, Electronic structure calculations with GPAW: a real-space implementation of the projector augmented-wave method, J. Phys-Condens. Mat. **22**, 253202 (2010).
